# Editorial: Going beyond the traditional tools of implementation science

**DOI:** 10.3389/frhs.2023.1343058

**Published:** 2023-12-21

**Authors:** Per Nilsen, Jeanette Wassar Kirk, Kristin Thomas

**Affiliations:** ^1^ Department of Health, Medicine and Caring Sciences, Linköping University, Linköping, Sweden; ^2^ School of Health and Welfare, Halmstad University, Halmstad, Sweden; ^3^ Department of Clinical Research, Hvidovre University Hospital, Hvidovre, Denmark; ^4^ Department of Health and Social Context, National Institute of Public Health, University of Southern Denmark, Odense, Denmark

**Keywords:** theories, models, frameworks, study design, strategies

**Editorial on the Research Topic** Going beyond the traditional tools of implementation science

## Introduction

Implementation science is evolving and novel approaches are required to account for the complexity of implementation processes. The Research Topic *Going Beyond the Traditional Tools of Implementation Science* called for papers presenting innovative approaches to advance our knowledge on implementation.

## Theories, models and frameworks

Research in implementation science employs three types of tools to understand and explain implementation and to close the research-practice gap. A crucial tool is the use of theories, models and frameworks (TMFs) to identify, describe and evaluate determinants (usually distinguished into barriers and facilitators), processes and outcomes of implementation. Five of the contributions concerned TMFs.

Birken et al. describe the development of the Organization Theory for Implementation Science (OTIS) framework which seeks to increase researchers' familiarity with organizational influences on implementation. Their paper describes the use of concept mapping and iterative consensus-building to identify six conceptually distinct domains, encompassing 70 constructs from nine organization theories. The domains reflect concepts that are central to organization theory, including, for example, autonomy and power, but which are less commonly addressed in implementation science.

Another perspective on organizational influences is provided by Scheuer. Translation theories take a process view that uses the sequence of events, activities and choices by “translators” (e.g., healthcare providers) to explain outcomes of implementation processes. According to the translational perspective, the spread of anything, e.g., a clinical guideline, is in the hands of people who may act in many different ways to modify or add to it. Contrasting with most implementation science TMFs, translation theories downplay the possibility to foresee what determinants may influence implementation.

Steerling et al. present a scoping review examining eight studies concerning trust when implementing AI systems in healthcare. Trust as a theoretical construct is rarely explicitly considered in the TMFs in implementation science but may be critical to understand AI systems implementation. The authors found that most studies had an individual perspective where trust was directed toward the AI technology. However, the review also included studies that focused on trust as relational between people within the context of the AI application.

Few determinant frameworks in implementation science account for the sustainment of evidence-based practices. Nadalin Penno et al. describe the Sustaining Innovations in Tertiary Settings (SITS) framework, which addresses determinants to sustainment specifically. They combined a systematic review and theory analysis of known sustainability TMFs with results from a case study using mixed methods to examine the ongoing use of an evidence-based practice in tertiary care. SITS consists of seven sustainability constructs, including innovation, adopters, leadership and management, inner context, inner processes, outer context, and outcomes.

Meza et al. present a different perspective on TMFs by showing how researchers can engage in a process of theorizing that draws on empirical data rather than treating existing theories as static products. Researchers who use TMFs deductively in studies usually fail to inductively modify theory based on their findings. The authors argue that theorizing can advance theory, thus contributing to improved explanation of implementation. They provide an example of how a theory theorizing can be constructed through developing causal explanations.

## Strategies

Another type of tool is the development and application of strategies for facilitating the implementation of evidence-based practices. These should ideally be matched to existing determinants to reduce barriers and harness facilitators to implementation. Three of the contributions focused on strategies.

Jones et al. used intervention mapping to identify and match strategies to barriers and to develop programmes to improve familial hypercholesterolemia (FH) care. The paper includes a scoping review and a parallel mixed method study using interviews and surveys. Barriers were found to exist for all components (identification, cascade testing and management) and all levels (patient, clinician and health system) of FH care. The authors listed strategies specific to FH care that others can adopt to their local context.

Stakeholder involvement is increasingly emphasized in implementation science. Woodward et al. describe the development of a consumer engagement implementation strategy called Consumer Voice (set of trust-building tools). The tools were developed in a multi-step human-centered design process in the context of a suicide prevention intervention in Arkansas. They are available online, consisting of slides, audiovisual content with written text and templates.

Ingvarsson et al. used applied behaviour analysis to understand and develop de-implementation strategies. The analysis focused on the unnecessary use of x-rays for knee arthrosis in a primary care centre. The analysis provided the basis for the development of a lecture and feedback meetings as two strategies to reduce this practice. The results were inconclusive but indicated a behaviour change in the desired direction.

## Research methodology

A third type of tool in implementation science is the research methodology used to investigate the process and outcomes of implementation efforts. Robust research methods must be used, and appropriate measures are needed to document the process and outcomes, including the effectiveness of various strategies. Four contributions addressed research methods and measures to study implementation.

Pinero de Plaza et al. present the development and testing of a novel evaluation method, the PROLIFERATE framework, which combines ecological (e.g., emergent system properties) and social logic models (study of individuals, groups and organizations) with the predominantly mechanistic logic of implementation science (i.e., bringing evidence-based interventions into practice). The paper describes examples of ongoing research to demonstrate how the framework can be used for co-designing innovations and evaluating implementation processes and outcomes.

Harvey et al. present a discussion paper advocating context-responsive study designs, i.e., designs that have high degree of adaptability and better align with the realities of implementation practice. The paper is based on workshop discussions among the authors and consultations with an international group of researchers and practitioners. The paper emphasizes the importance of engagement between implementation researchers and practitioners and acceptance of more flexible study designs.

Swindle et al. propose Evidence-Based Quality Improvement (EBQI) as an example of a method to achieve community engagement in implementation research and practice. EBQI expands on quality improvement and involves a deliberative and partnered process emphasizing a partnership between research and practice. The method involves activities such as selection and tailoring of implementation strategies and iterative adaptations of innovations.

Fixsen et al. emphasize the need for commonly used measures of implementation processes and outcomes. They argue that lack of valid measures has hindered the advancement of knowledge on implementation. The paper presents a literature review on measures on implementation variables resulting in 32 articles including measures of 23 implementation variables such as implementation fidelity.

## Discussion

The papers on the three tools of TMFs, strategies and research methodology in implementation science present novel approaches that strive to capture the complex and dynamic nature of real-world implementation. The field has medical origins in the evidence-based movement, yet real-world implementation has been found to be highly context-dependent. The themes of the papers exemplify the balancing act within the field whereby context-specific studies are needed as well as studies that produce findings that can be generalized across contexts for more broadly applicable conclusions. This Research Topic points to the importance of a social science perspective to understand how humans and organizations act and interact in their social environment.

